# Oldest record of Trimeniaceae from the Early Cretaceous of northern Japan

**DOI:** 10.1186/1471-2148-8-135

**Published:** 2008-05-08

**Authors:** Toshihiro Yamada, Harufumi Nishida, Masayoshi Umebayashi, Kazuhiko Uemura, Masahiro Kato

**Affiliations:** 1 Division of Life Sciences, Graduate School of Natural Science and Technology, Kanazawa University, Kanazawa 920-1192, Japan; 2 Faculty of Science and Engineering, Chuo University, Tokyo 112-8551, Japan; 3 Department of Geology and Paleontology, National Museum of Nature and Science, 3-23-1 Hyakunincho, Tokyo 169-0073, Japan; 4 Department of Botany, National Museum of Nature and Science, Tsukuba 305-0005, Japan

## Abstract

**Background:**

Molecular phylogenetic analyses have identified Trimeniaceae, a monotypic family distributed only in Oceania, as among the earliest diverging families of extant angiosperms. Therefore, the fossils of this family are helpful to understand the earliest flowering plants. Paleobotanical information is also important to track the historical and geographical pathways to endemism of the Trimeniaceae. However, fossils of the family were previously unknown from the Early Cretaceous, the time when the angiosperm radiated. In this study, we report a seed from the late Albian (ca. 100 million years ago) of Japan representing the oldest known occurrence of Trimeniaceae and discuss the character evolution and biogeography of this family.

**Results:**

A structurally preserved seed was collected from the early Late Albian Hikagenosawa Formation of the Yezo Group, which was deposited in palaeolatitudes of 35 to 40°N. The seed has a multilayered stony exotesta with alveolate surface, parenchymatous mesotesta, and operculate inner integument, which are characteristic to extant trimeniaceous seeds. However, the seed differs from extant seeds, i.e., in its well-developed endosperm and absence of antiraphal vascular bundle. Thus, the seed would be a new genus and species of Trimeniaceae.

**Conclusion:**

The fossil seed indicates that seed coat characters were conserved for 100 million years or more in Trimeniaceae. It also suggests that the antiraphal vascular bundle and perispermy originated secondarily in Trimeniaceae as previously inferred from the phylogeny and character distribution in the extant basalmost angiosperms. The fossil seed provides the first evidence that Trimeniaceae was distributed in a midlatitude location of the Northern Hemisphere during the Early Cretaceous, when angiosperms radiated extensively, supporting a hypothesis that the extant austral distribution is relict.

## Background

Trimeniaceae is a small family consisting of only the genus *Trimenia*, with eight species known from Celebes to eastern Australia and the southwest Pacific [1,2]. *Trimenia *consists of shrubs and lianas that have unisexual or bisexual flowers with numerous tepals and stamens and a single carpel [1-4]. A solitary pendant ovule is enclosed in the ovary and develops into a seed with a stony seed coat contained in a berry [1,4-7]. The family was placed in the Laurales based on floral structures, such as the single carpel and ovule per flower [3,4], but molecular phylogenetic studies have identified it as one of the earliest diverging families of extant angiosperms, along with the Amborellaceae, Nymphaeales [Cabombaceae and Nymphaeaceae], Hydatellaceae, Austrobaileyaceae, and Illiciaceae [8-11]. Thus, clarifying the primitive character states of Trimeniaceae would be helpful in understanding the earliest flowers. In particular, information on key innovations of angiosperms, for example, fruit, bitegmic seeds, and endosperm, would provide clues to solving the origin of angiosperms, a central mystery of plant evolution [12,13].

Great efforts have been made to find evidence of early angiosperms from the Early to mid-Cretaceous, when the angiosperms radiated extensively [14-20]. The early emergence of Nymphaeales [18,21] and Illiciaceae [16] in the fossil record partly supports the phylogenetic framework inferred from molecular data. However, direct evidence that the other earliest diverging families emerged in the early phase of angiosperm radiation [20] has not been found, although paleobotanical records [16,18], as well as phylogeny [8-11], imply that these families should have diverged by the mid-Cretaceous [22].

From the austral distribution of Trimeniaceae, and the similar distribution of Amborellaceae and Austrobaileyaceae [1], neobotanists have inferred the Gondwana origin of these families [23,24], but accumulated palynological data have indicated that a different explanation is needed for this distribution. These data indicate that angiosperms originated in low paleolatitudes (20°N to 20°S) no later than the Hauterivian, Early Cretaceous, about 132 million years ago [25,26] and spread toward the poles over time [26-28]. Pollen records provide temporal and spatial distribution patterns of angiosperms, with the implication that the Trimeniaceae could have migrated to Laurasia, a continental mass in the Northern Hemisphere. However, pollen is difficult to assign to extant families due to the frequent convergent evolution of pollen morphology, such as the number and position of apertures [20,29]. Moreover, extant *Trimenia *pollens are eurypalynos with regard to the number of aperture even in a same species [29], obscuring diagnostic pollen characters in familiar level. Thus, findings from other reproductive structures, such as flowers, fruits, and seeds, are needed to confirm the past existence of the Trimeniaceae in the Northern Hemisphere.

We report the oldest seed of Trimeniaceae from the Early Cretaceous Yezo Group in Hokkaido, northern Japan, and discuss character evolution and biogeography of the Trimeniaceae.

### Geological settings and other paleobotanical data of collection site

**Figure 1:**
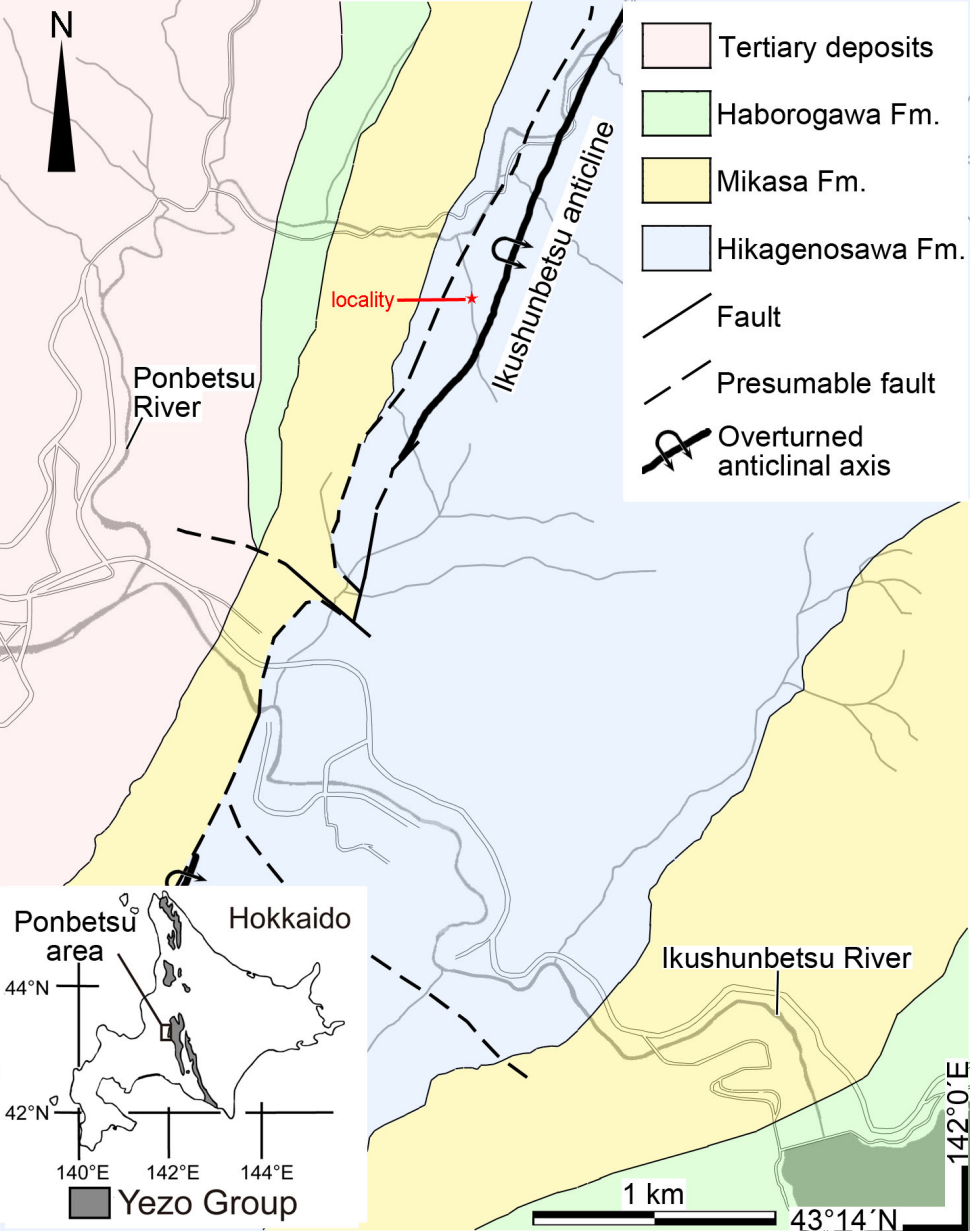
**Geological map of Ponbetsu area and locality of the trimeniaceous seed**. Ponbetsu area are boxed in large-scale map of Hokkaido on the left-bottom corner. Geological map is redrawn from Narita et al. 31.

A structurally preserved seed was found within a calcareous siltstone nodule collected at Pombetsu, Mikasa City, Hokkaido, Japan (Figure 1). The Hikagenosawa Formation of the Yezo Group outcrops in the area and contains ammonoids indicating a date in the early Late Albian [30,31]. Palaeomagnetic studies show that these sediments were deposited in palaeolatitudes of 35 to 40°N and that the sedimentary basin was located on the eastern side of Laurasia [30,32]. Because the formation mainly consists of offshore sediments, there are few palaeobotanical records within it [33-35], but a subtropical climate with dry seasons seems to have prevailed in the area, as suggested by the occurrence of a cheirolepidiaceous conifer [34] and a variety of ephedroid palynomorphs [33].

## Results

**Figure 2:**
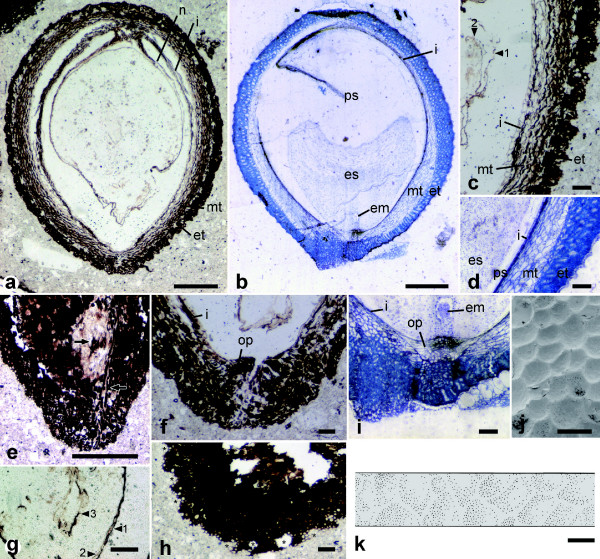
**Fossil and extant seeds of Trimeniaceae share many morphological characters**. Section of fossil (a, c, e-h) and seed of *Trimenia moorei *(b, d, i); surface of *T. moorei *(j) and fossil (k) seed. Close-up (c) of the micropylar part of (a); (g) is near apex of the nucellus. em, embryo; es, endosperm; et, exotesta; i, inner integument; mt, mesotesta; op, operculum; ps, perisperm. (c, g) arrowheads indicate the nucellar epidermis (1), endosperm membrane (2) and embryo (3). (e) Arrows show tracheids and fibers observed in the hilum. (h) Asterisks indicate areoles seen in nearly paradermal sections of the surface. Scale bars in a and b, 500 μm; e, 200 μm; others, 100 μm.

The seed is ellipsoid, 3 mm long and 2.2 mm thick. It has an inner integument and an outer seed coat (testa) (Figures 2a,c,e). The micropyle is formed by both inner integument and testa (endostome and exostome, respectively) and adjacent to the hilum, which contains tracheids and sclerenchymatous fibers, i.e., the seed is anatropous (Figures 2e,f). These tracheids and fibers are found only in sections through the raphal side, thus the vascular bundle does not extend to the antiraphal side beyond the chalaza (Figure 2e). The inner integument is almost crushed, except in the endostomic region, where the inner integument is thickened to form the operculum (Figures 2c,f). The testa consists of an outer (exotesta) and inner (mesotesta) part. The exotesta comprises one to five layers of isodiametric cells, while the mesotesta is made up of four to five layers of longitudinally elongated cells. The exotesta is lignified to give mechanical strength to the seed coat (Figure 2c). In a nearly tangential section through the seed surface, there are polygonal openings in the exotesta, indicating the presence of polygonal areoles on the exotestal surface (asterisks in Figure 2h). This configuration was further examined by reconstructing a three-dimensional image of the seed surface by compiling serial peel sections, revealing that the exotestal surface is alveolate (Figure 2k). Inside the inner integument are three membranous structures. The outermost structure, connected to the chalaza, is the nucellar epidermis (Figure 2a and arrowheads in Figures 2c,g). The innermost structure is observed only in sections through the center of the seed and encircles an area 200 μm in length and 40 μm in width (arrowhead 3 in Figure 2g).

## Discussion

### Relationships of the fossil seed

Although seed structures have not been examined in all *Trimenia *species, available information on four species (*T. moorei*, *T. neocaledonica*, *T. papuana*, and *T. weinmanniifolia*) suggests that Trimeniaceae is uniform family in regard of ovule and seed structures [1,4-7]. The seed of extant *Trimenia *is characterized by the testa, consisting of a lignified multilayered exotesta with an alveolate surface and a nonlignified multilayered mesotesta (Figures 2b,d,i,j) [1,4,7]. Although many families possess seed coats with a hardened exotesta, none other than Trimeniaceae is known to possess a multilayered exotesta [1,4,7,36]. The seeds of some taxa are similar in appearance to the Trimeniaceae seed in that the testa consists of outer sclerotic and inner non-sclerotic cell layers. These taxa include the genus *Nuphar *(Nymphaeaceae) [37,38], the families Buxaceae [36], Simmondsiaceae [39], Melianthaceae [40], Myrtaceae [36,41], and Theaceae [36], and the order Sapindales [36,42]. However, in these taxa, the stony outer layer is composed of a single-layered columnar exotesta and the outer part of the isodiametric mesotestal cells, whereas the exotesta of *Trimenia *is composed uniformly of sclerotic isodiametric cells, which are derivatives of the outer epidermis of the outer integument [7]. Thus, to our knowledge, no other seeds can be compared to the fossil seed.

The fossil seed is not distinguishable from that of extant *Trimenia *in terms of shape, size, anatropy, bitegmy, endo and exostomic micropyle, crushed inner integument with an operculum, lignified multilayered exotesta composed of isodiametric cells, alveolate surface of the exotesta, or nonlignified multilayered mesotesta (Figure 2). These remarkable similarities unequivocally indicate the close affinity of the fossil seed to the Trimeniaceae. The fossil seed differs from *Trimenia *seed in the absence of antiraphal vascular bundle (Figure 2e) and in the size of membranous structures surrounding the nucellus (Figures 2c,g; see below for details). Thus, a new genus of Trimeniaceae should be assigned to the fossil seed. The seed also differs from *Trimenia *seed in having a stalklike structure at the nucellar base (Figure 2a). This stalklike structure might represent a diagnostic feature of the seed, but the structure could be artificially formed by shrinkage.

### Divergence time of Trimeniaceae

Molecular phylogeny indicates that the Trimeniaceae diverged after the Nymphaeales and before the Illiciaceae [8-11]. Thus, diversification of the Trimeniaceae in the Early to earliest Late Cretaceous (from 125 to 90 million years ago) is indicated [22] by the Late Barremian flower of the Nymphaeales from Portugal [18] and the Cenomanian to Turonian seeds of the Illiciaceae from Kazakhstan [16]. Although Trimeniaceae-like triporate or polyforate pollen was collected in the Albian to Cenomanian of Brazil [43,44], the Barremian of Portugal [15,17] and the Campanian to Maastrichitian (83–65 million years ago) of Australia and Antarctica [44,45], the possibility could not be ruled out that these are assigned to other families [20,29,44,46]. *Longstrethia varidentata*, a foliar species reported from the Cenomanian of Nebraska, is similar to leaves of Trimeniaceae in presence of an intermarginal vein, but *L. varidentata *would represent a stem-group taxon of Trimeniaceae-Illiciaceae clade because some characters are also shared with Illiciaceae [47]. Therefore, no unequivocal record of Trimeniaceae in the Cretaceous existed. The fossil seed provides the first unequivocal evidence of Trimeniaceae 100 million years ago that fills the gap between molecular data and paleobotanical records.

### Character evolution in Trimeniaceae

The fossil seed coat structures are strikingly similar to those of *Trimenia *(Figure 2). Although these characters were acquired 100 million or more years ago and are conserved in Trimeniaceae, the mode of nutrient storage of the fossil seed would have differed considerably from that of the extant *Trimenia*. The *Trimenia *embryo is enclosed by the endosperm, which is further surrounded by the perisperm, an additional storage tissue (Figures 2d,i) [4,6,7]. However, the fossil seed has two membranous structures inside the nucellus, and the area encircled by the inner structure corresponds in size to the dormant mature embryo of extant Trimeniaceae (Figures 2g,i). If this comparison is correct, the outer structure may be comparable to the endosperm membrane. In albuminous seeds without a perisperm, the epidermis of the nucellus is either completely disintegrated by the enlarged endosperm or vestigially retained as a crushed layer, whereas in perispermous seeds, it is retained as the epidermis of the perisperm [6,7,36]. The fossil has a relatively well preserved nucellar epidermis, which may imply the presence of a perisperm. The putative endosperm in the fossil occupies a large area inside the nucellus, and thus the endosperm would have played a major storage role. Among the earliest families to diverge, only Nymphaeales [38], Hydatellaceae [48], and Trimeniaceae [4,6,7] have a diploid maternal perisperm, which occupies a larger area than the diploid or polyploid fertilized endosperm. This character variation implies that the storage function was largely taken over by the perisperm secondarily [49,50] and that the fossil seed may have been on the way toward perispermy.

The fossil seed vasculature is different from that of extant *Trimenia*. In *Trimenia*, the vascular bundle supplied from the fruit wall enters the raphe of the seed and extends to the antiraphal side beyond the chalaza [4]. Lack of antiraphal vascular bundles in the fossil seed suggests that the antiraphal bundle is a derived character in the Trimeniaceae. This is consistent with the hypothesis, based on character distribution in extant basalmost angiosperms, that the antiraphal bundle is a derived character in the angiosperm seed [6,51].

So far, we know only the seed of this trimeniaceous fossil, but the areoles sculptured in the seed surface may imply that the fossil seed was contained in a berry because the pressure of berry endocarp cells forms areoles on seeds of extant *Trimenia *[1-7].

### Biogeography

Extant Trimeniaceae species are distributed in eastern Australia and an island chain stretching from Celebes to the Moluccas, New Guinea, New Caledonia, Fiji, Samoa, and the Marquesas [1,2,4]. Along with the Trimeniaceae, other basalmost families (Amborellaceae, Hydatellaceae, Austrobaileyaceae) and many eumagnoliid families (e.g., Degeneriaceae, Monimiaceae, Winteraceae) now grow only in derivative fragments of Gondwanaland [1,4,52]. Contrary to their extant austral distribution, palynological records suggest that early angiosperms originated in the low latitudes [25,26] and migrated both northward and southward [26-28]. Thus, the paleobotanical data indicate that the current austral distributions do not reflect the cradle of angiosperms but the area of conservation [26-28]. At the same time, the poleward migration model predicts the past occurrence of these basalmost angiosperm families in the Northern Hemisphere. Some pollen grains, tentatively assigned to the Amborellaceae [53] or Trimeniaceae [15,17,20], are reported from the Northern Hemisphere, but familial assignations of pollen are difficult [20,29,46]. The fossil seed reveals that Trimeniaceae occurred in the eastern margin of Laurasia in the Northern Hemisphere during the Albian period and indicates a reduction to the relict area subsequent to the hypothesized bipolar migration.

## Conclusion

Fossil seed of Trimeniaceae is described from the Early Cretaceous (ca. 100 million years ago) Yezo Group in Hokkaido, northern Japan. The seed, which is the oldest yet found for the family, indicates; 1) Some seed coat structures of Trimeniaceae have been conserved for about 100 million years, including the multilayered stony exotesta with alveolate surface, parenchymatous mesotesta, and operculate inner integument. 2) The secondary origins of the perisperm and antiraphal vascular bundle. 3) Trimeniaceae was distributed in a midlatitude location of the Northern Hemisphere during the Early Cretaceous, when angiosperms radiated extensively, supporting that the extant austral distribution is relict.

## Methods

Materials (*Yamada 001002*) and methods for plastic sections and SEM micrograph of *Trimenia moorei *(extant) were described previously [7]. The fossil seed was sectioned in planes tangential to the raphe, and the extant seed was sectioned in planes parallel to the raphe. A part of the fossil seed, 1.5 mm thick, was cut into 38 successive peel sections, i.e., one section is ca. 40 μm thick. For reconstruction, the outline of the seed surface was traced, and surface views were compiled manually by overlying successive camera lucida drawings. The slides of serial sections and the nodule containing the seed are stored in the National Museum of Nature and Science, Tokyo, Japan, as NSM-PP-9176.

## Authors' contributions

TY performed the field survey, found the fossil and collected data on the fossil and extant seeds. HN performed the field survey and provided facilities for making peel sections. MU drew the reconstruction of the fossil. KU collected data on fossils and provided facilities for making peel sections. MK organised the study and collected data on extant seeds. All authors discussed the results and commented on the manuscript.
